# The Symbiotic Continuum Within Ticks: Opportunities for Disease Control

**DOI:** 10.3389/fmicb.2022.854803

**Published:** 2022-03-17

**Authors:** Sabir Hussain, Nighat Perveen, Abrar Hussain, Baolin Song, Muhammad Umair Aziz, Jehan Zeb, Jun Li, David George, Alejandro Cabezas-Cruz, Olivier Sparagano

**Affiliations:** ^1^Department of Infectious Diseases and Public Health, Jockey Club College of Veterinary Medicine and Life Sciences, City University of Hong Kong, Kowloon, Hong Kong SAR, China; ^2^Department of Biology, College of Science, United Arab Emirates University, Al Ain, United Arab Emirates; ^3^Department of Epidemiology and Public Health, University of Veterinary and Animal Sciences, Lahore, Pakistan; ^4^School of Natural and Environmental Sciences, Newcastle University, Newcastle upon Tyne, United Kingdom; ^5^Anses, INRAE, Ecole Nationale Vétérinaire d’Alfort, UMR BIPAR, Laboratoire de Santé Animale, Maisons-Alfort, France

**Keywords:** tick microbiota, tick symbiont, symbiont-pathogen interactions, tick physiology, tick-borne diseases ecology, tick continuum

## Abstract

Among blood-sucking arthropods, ticks are recognized as being of prime global importance because of their role as vectors of pathogens affecting human and animal health. Ticks carry a variety of pathogenic, commensal, and symbiotic microorganisms. For the latter, studies are available concerning the detection of endosymbionts, but their role in the physiology and ecology of ticks remains largely unexplored. This review paper focuses on tick endosymbionts of the genera *Coxiella*, *Rickettsia*, *Francisella*, *Midichloria*, and *Wolbachia*, and their impact on ticks and tick-pathogen interactions that drive disease risk. Tick endosymbionts can affect tick physiology by influencing nutritional adaptation, fitness, and immunity. Further, symbionts may influence disease ecology, as they interact with tick-borne pathogens and can facilitate or compete with pathogen development within the vector tissues. Rickettsial symbionts are frequently found in ticks of the genera of *Ixodes, Amblyomma*, and *Dermacentor* with relatively lower occurrence in *Rhipicephalus, Haemaphysalis*, and *Hyalomma* ticks, while *Coxiella*-like endosymbionts (CLEs) were reported infecting almost all tick species tested. *Francisella*-like endosymbionts (FLEs) have been identified in tick genera such as *Dermacentor, Amblyomma, Ornithodoros, Ixodes*, and *Hyalomma*, whereas *Wolbachia* sp. has been detected in *Ixodes, Amblyomma*, *Hyalomma*, and *Rhipicephalus* tick genera. Notably, CLEs and FLEs are obligate endosymbionts essential for tick survival and development through the life cycle. American dog ticks showed greater motility when infected with *Rickettsia*, indirectly influencing infection risk, providing evidence of a relationship between tick endosymbionts and tick-vectored pathogens. The widespread occurrence of endosymbionts across the tick phylogeny and evidence of their functional roles in ticks and interference with tick-borne pathogens suggests a significant contribution to tick evolution and/or vector competence. We currently understand relatively little on how these endosymbionts influence tick parasitism, vector capacity, pathogen transmission and colonization, and ultimately on how they influence tick-borne disease dynamics. Filling this knowledge gap represents a major challenge for future research.

## Introduction

Ticks are hematophagous, obligate ectoparasites of terrestrial vertebrates such as amphibians, reptiles, birds, mammals, and humans (Black and Piesman, 1994; Estrada-Peña and Jongejan, 1999; Stafford et al., 2007; Anderson and Magnarelli, 2008). Ticks play a significant role in transmitting infectious diseases (Hussain et al., 2021b; Perveen et al., 2021b), and are competent vectors of a wide range of pathogens affecting animal and human health globally (de la Fuente et al., 2017; Hussain et al., 2021a,c). They are the most important vectors among those transmitting vector-borne pathogens to animals, and the second, after mosquitoes, for pathogens with impact for human health (de la Fuente et al., 2017; Hussain et al., 2021a,c). As a taxonomic group, ticks are well-adapted to a wide range of climatic conditions, thriving in tropical, temperate and even subarctic habitats (Anderson and Magnarelli, 2008). Ticks (Acari: Ixodida) are divided into three families: Ixodidae, Argasidae, and Nuttalliellidea (Anderson and Magnarelli, 2008; Perveen, 2021), and almost 28 tick species have been reported to transmit pathogens to humans (Anderson and Magnarelli, 2008). The prevalence of many tick-borne pathogens, such as *Babesia*, *Theileria*, and *Borrelia* has increased in recent years due to climate change and other anthropogenic factors such as land use change, deforestation, urbanization, global travel, and trade (Rochlin and Toledo, 2020). Ticks acquire these pathogens during blood feeding on an infected vertebrate host (Raffel et al., 2014). Some colonize the tick midgut (Raffel et al., 2014), and migrate to the salivary gland from where they are transmitted *via* tick feeding to a new host. Although less common, direct transmission of some pathogens (e.g., *Borrelia afzelii*) from tick midguts has also been reported (Pospisilova et al., 2019).

Some bacteria, mainly present in the Malpighian tubules or ovaries of ticks, act as endosymbionts and are harmless to vertebrate hosts (Rowley et al., 2004). Obligate symbionts are indispensable for tick survival and fitness, frequently transmitted from adult females to their offspring, while some facultative symbionts as Cardinium or Spiroplasma are reproductive parasites and have major impacts on reproduction of arthropods (Perlman et al., 2006). Bacterial symbionts that are vertically transmitted from mother to offspring increase the fitness of the tick by directly enhancing the reproductive capacity of the vector, which in turn facilitates and increases the symbiont survival and persistence across arthropod hosts generations (Brouqui et al., 1993). However, whilst our current understanding of the life cycle of these symbionts is typically framed within vertical transmission only, horizontal transmission has been observed in a number of cases. For example, evidence of horizontal transmission has been reported in tick symbionts such as *Midichloria* (Bazzocchi et al., 2013), *Coxiella* (Shivaprasad et al., 2008; Seo et al., 2016; Mioni et al., 2020; Kobayashi et al., 2021) and *Arsenophonus* (Edouard et al., 2013) strains (Bonnet et al., 2017). Horizontal transmission is also common in symbionts of other arthropods such as parasitoid wasps (Parratt et al., 2016). Exposure of vertebrate hosts to tick symbionts have been regarded as evidence of the pathogenic potential of symbionts (Shivaprasad et al., 2008). Tick-host-symbiont relationships have been thus described as a continuum of “mutualism,” “commensalism,” or “parasitism” (Bonnet et al., 2017). But the role of vertebrate hosts in the ecology and life cycle of tick symbionts remains poorly understood due to a paucity of research in this area.

Other bacteria, that are neither human pathogens nor strict symbionts, can be regarded more generally as tick microbiota (Wu-Chuang et al., 2021a). Within the text, “microbiota” only refers to the microbes themselves, whereas “microbiome” refers to the microorganisms and their genes. Cowdry (1925) first recognized the relationship between ticks and their microbiome at the beginning of the twentieth century. His work reported *Rickettsia*-like bacteria in the ovaries, eggs, Malpighian tubules, and intestinal epithelial cells of 16 different tick species (Cowdry, 1925). The first tick microbiome study to employ next-generation sequencing (NGS) was published in 2011 by Andreotti et al. (2011). Since then, an increasing number of NGS studies have been used to characterize the tick microbiome, allowing for a broader view of its taxonomic composition in several tick species (Wu-Chuang et al., 2021a). In addition to pathogens and obligate endosymbionts, ticks carry commensal non-pathogenic microorganisms that complement tick nutrition and interact with tick-borne pathogens, affecting tick fitness and vector competence (Wu-Chuang et al., 2021a). Due to the complex nature of colonization of tick microbiota, the interaction between endosymbionts and pathogens is sometimes hard to understand, with these associations potentially affecting tick physiology and ecology (Bonnet et al., 2017). Nevertheless, it is known that changes in microbial communities can modulate vector competence by decreasing, for example, *Borrellia burgdorferi* colonization in *Ixodes scapularis* larvae (Narasimhan et al., 2014). Similar examples have been previously reviewed (de la Fuente et al., 2017; Wu-Chuang et al., 2021a). Not only the microbiota modulates pathogen colonization, but pathogen entry within the tick midgut milieu trigger changes in the microbial communities (Abraham et al., 2017). For example, *Anaplasma phagocytophilum* infection in ticks disturbs the gut microbiota, increasing the presence of *Pseudomonas* and reducing the abundance of *Rickettsia* and *Enterococcus* (Abraham et al., 2017), which was associated with a reduction of bacterial biofilms in tick midguts (Heisig et al., 2014; Abraham et al., 2017) and also a decreased representation of biofilm synthesis pathways in the tick microbiome (Estrada-Peña et al., 2020b). *B. burgdorferi* infection increased the expression of the tick protein PIXR, which alter the gut microbiome, metabolome and immune responses and facilitates *B. burgdorferi* infection and molting of larvae (Narasimhan et al., 2017). Accordingly, it has been proposed that the relation between pathogens and microbiota is bidirectional (Cabezas-Cruz et al., 2018).

Developing a comprehensive understanding of the role of microbiota in governing the physiology and ecology of ticks, and its interaction with tick-borne pathogens could prove highly beneficial for devising new strategies to control and prevent tick-borne diseases. This review paper describes and discusses the most common symbiotic and endosymbiotic tick-microbiota relationships, highlighting their relevance for tick physiology and ecology. We focus primarily on published studies providing species-level resolutions for symbiont characterization, most often through PCR and whole genome sequencing. We also included studies using metagenomics approaches and NGS, a technique that has revolutionized microbiome research (Kulski, 2016), allowing for high throughput and high-resolution assessment of the assortment of circulating microorganisms in tick vectors. Thanks to these advances, it is now relatively straight-forward to characterize tick endosymbiont assemblages to species levels, with the resulting growing body of published work in this area prompting this review.

## The Symbiotic Continuum in Ticks

Non-pathogenic microbes found in ticks can classified as commensals and endosymbionts. Within commensals, a highly variable group of bacterial taxa, referred to as microbiota, have been described (Narasimhan and Fikrig, 2015). The microbiota composition is under the influence of several factors including the tick species, physiological stress by environmental traits, blood-meal, host species, tick immunity, and developmental stage (Narasimhan and Fikrig, 2015). Despite the variability of bacterial taxa associated to ticks, bacterial communities in the tick microbiome are functionally redundant (Estrada-Peña et al., 2020a). For example, the microbiome of *I. scapularis* larvae and nymphs shared 80 taxa (24.6%, total 324), while out of 342 predicted metabolic pathways, 82.7%, were shared by all the tick samples (Estrada-Peña et al., 2020a). Furthermore, the *I. scapularis* microbiota exposed to pathogen infection, antimicrobial peptide or anti-tick host immunity shared a very reduced taxonomic core of 61 bacterial genera (7.4%, total 821), while the majority (i.e., 381) of the metabolic pathways (87.2%, total 437) were identified in all the samples (Estrada-Peña et al., 2020b), Also, despite high temperature altered the structure of the microbial community in *I. scapularis* (Thapa et al., 2019; Wu-Chuang et al., 2021b) four keystone taxa found across the temperature gradient, and their directly connected neighbors, contributed to more than 99% of the predicted pathways regardless the incubation temperature (Wu-Chuang et al., 2021b). This suggests that the tick microbiome is a stable source of metabolic functions with potential implications for tick physiology. A mechanism can then be proposed by which ticks are permissive to colonization by environmental bacteria fulfilling a specialized set of functions.

**FIGURE 1:**
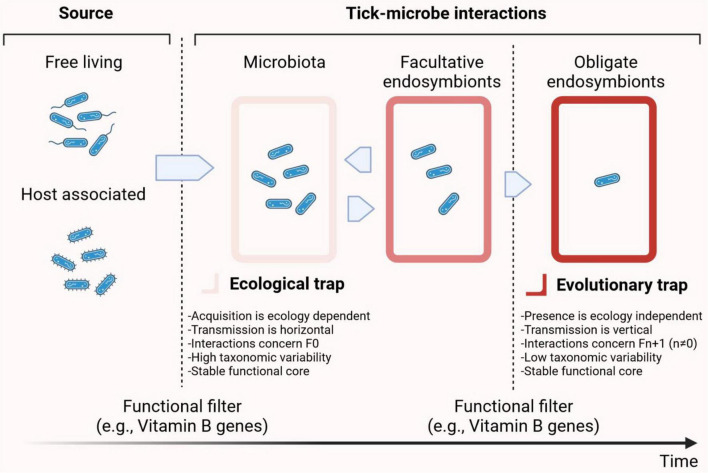
Symbiotic continuum in ticks.

Intriguingly, metabolic pathways associated with nutritional complementation by symbionts are broadly distributed in the tick microbiome. For example, the presence of vitamin B synthesis genes in *Francisella* or *Coxiella* symbionts (see details below) compensate for the shortage of vitamin B in the blood meal (Duron et al., 2018). However, vitamin B synthesis genes are not restricted to symbiotic bacteria of the genera *Francisella* or *Coxiella*, but are widely distributed throughout several bacterial genera of tick microbiota (Obregón et al., 2019). In addition, considering the high diversity of genes and metabolic pathways encoded in the genomes of tick microbiota bacteria, we hypothesize that the contribution of bacteria to tick physiology and survival could extend well beyond vitamin B supplementation (Obregón et al., 2019; Estrada-Peña et al., 2020b). Indeed, the metabolic pathways associated with the tick microbiome include processes as diverse as amino acid, antibiotic, pyrimidine, lipid, and amino sugar metabolism (Obregón et al., 2019). Thus, an ecological-to-evolutionary continuum could be proposed in which environmental, free-living and/or host-associated, bacteria colonize tick tissues and later, under certain conditions, establish a symbiotic relationship with the tick host (Figure 1).

## Impact of Symbionts on Tick Physiology

The recent emphasis on tick microbiome research has provided a new dimension of the microbes carried by ticks. Scientists are investigating symbiotic interactions and their impact on invertebrate host’s physiology (Guizzo et al., 2017). Ticks usually harbor two types of endosymbionts. The first group includes obligate mutualistic symbionts, transmitted from mother to offspring, which are essential for the development and growth of their host, supporting various functions such as nutrition (Wernegreen, 2012), and behavior (Zhong et al., 2021). The second group comprises facultative symbionts, which are not as important as their obligate counterparts in ensuring host survival (Ohtaka and Ishikawa, 1991). Nevertheless, facultative symbionts can also affect their host, where, for example, those found within reproductive organs can manipulate reproduction and physiology by inducing parthenogenesis and cytoplasmic incompatibility (Cordaux et al., 2011). *Coxiella*-like endosymbionts (CLEs) preferentially colonize the ovaries and Malpighian tubules of ticks (Cibichakravarthy et al., 2022). The colonization of CLEs in the ovaries of ticks promote reproduction and developmental processes and assists its maternal transmission to the offspring (Klyachko et al., 2007). The dense colonization of CLEs in Malpighian tubules assist in its nutritional role as Malpighian tubules are engaged in nitrogenous products excretion and osmoregulation (Dhooria, 2016; Cibichakravarthy et al., 2022). CLEs may recycle arthropods’ hemolymph metabolites to synthesize vitamin B (Cibichakravarthy et al., 2022). Further, vitamin B synthetic pathway enzymes have been detected significantly more abundant in Malpighian tubules as compared to ovaries (Cibichakravarthy et al., 2022) in *Rh. sanguineus*. FLEs are maternally inherited symbionts of ticks (Gerhart et al., 2016; Kumar et al., 2021). FLE was identified as an obligate nutritional mutualist in the life cycle of *Ornithodoros moubata* through experiments and synthesizes B vitamins (Duron et al., 2018) that are deficient in the ticks’ blood meal. The elimination of *Francisella* F-Om alters ticks’ life history traits (Duron et al., 2018). Comparison of the metabolic pathways present in FLE-Am to that of CLEAA showed that the metabolic capability of FLE-Am is extensive than CLEAA, for example, FLE-Am can produce heme in addition to cofactors (except thiamine) synthesized by CLEAA (Gerhart et al., 2016). Further, FLE provides cysteine, which found in fewer concentrations in blood meal and can synthesize glutamine from glutamic acid and ammonia, so recycling cellular ammonia waste to useful products. Therefore, FLE-Am has superior biosynthetic capability as compared to CLE (Gerhart et al., 2016). *Wolbachia* have been shown to influence the reproduction of infected insects in various ways, including parthenogenesis, male killing, cytoplasmic incompatibility, and feminization (Hurst et al., 1999; Stouthamer et al., 1999). *Candidatus* Midichloria mitochondrii is widespread in various ixodid ticks (Epis et al., 2008) was found abundant in unfed and semi-engorged *Ixodes ricinus* females that enhance the host fitness by supplying essential nutrients (Olivieri et al., 2019). In general, symbiotic partners enter into an evolutionary spiral that leads to an irreversible codependence with associated risks (Bennett and Moran, 2015), while the microbiota allows for more flexible tick-microbe interactions determined by and adapted to ecological conditions. Tick microbiota and its interactions with the tick and pathogens have been recently revised (Wu-Chuang et al., 2021a). In the sections below, we collate our current understanding about tick endosymbionts of the genera *Coxiella*, *Rickettsia*, *Francisella*, *Midichloria*, and *Wolbachia*, as summarized in Supplementary Table 1.

### Coxiella

*Coxiella*-like endosymbionts is a obligate intracellular, maternally inherited bacterium, and found in high prevalence in tick populations (Duron, 2015). It usually engaged in mutualistic interactions with tick hosts (Brenner et al., 2021; Nardi et al., 2021). Several tick genera have been found to harbor CLE, including *Amblyomma* (Clay et al., 2008), *Dermacentor* (Jiao et al., 2021), *Haemaphysalis* (Lee et al., 2004), *Rhipicephalus* (Špitalská et al., 2018), *Ixodes* (Kurtti et al., 2002; Schabereiter-Gurtner et al., 2003; Špitalská et al., 2018), *Ornithodoros* (Duron, 2015), and *Carios* (Reeves et al., 2005). Douglas (2007) proposed that the benefits derived from the symbiotic relationship with CLE is limited in ticks, but it is considered that they play an important role in the synthesis of several nutrients that are required by their tick hosts (Moran et al., 2003; Douglas, 2007). As strict hematophagous parasites, ticks rely on the nutritional composition of host blood to support their metabolism (Heisig et al., 2014). Nutritional deficiencies in the blood, such as the lack of sufficient amounts of vitamin B, provide the basis for symbiotic partnerships with bacteria that synthesize vitamin B. This is a common challenge for arthropods feeding on blood, which frequently carry bacteria that synthesize vitamins and thus contribute to the fitness of their tick host (Beard et al., 2001; Akman et al., 2002; Wu et al., 2006). Further, Zhong et al. (2021) demonstrated that reduction in the abundance of the CLE in *Haemaphysalis longicornis* (CHI) decreases blood intake in ticks. It was found that reduced CLE abundance reduces serotonin biosynthesis that is essential in regulating tick-feeding activity. Researcher determined that providing tetracycline-treated ticks with the CHI-derived tryptophan precursor chorismate restores the feeding defect. Further, its increased level in the synganglion and midgut promotes tick feeding. Herbicide glyphosate treatment suppresses blood-feeding behavior in ticks by inhibition of CHI chorismate biosynthesis. Therefore, -CHI regulate tick feeding activity (Zhong et al., 2021).

The genome size of CLE is smaller than that of *Coxiella burnetii*, an obligate intracellular bacterium that causes Q fever in humans and animals (Stein and Raoult, 1992), and they are deficient in certain proteins including *recN* gene product that involved in DNA repair along with that *Amblyomma* bacterium may also lack the DNA repair function provided by *recN*, hence it impairs DNA replication (Jasinskas et al., 2007). This evidence indicated that this gene loss in this bacterium is the same as *C. burnetii* but also that it has a reduced genome, a common feature of obligate endosymbionts of invertebrates (Moran and Wernegreen, 2000; Jasinskas et al., 2007).

*Coxiella*-like endosymbionts have evolved as endosymbionts, being found at greater loads in the *A. americanum* tick salivary glands (Klyachko et al., 2007), where presence has been reported as necessary for ensuring the survival of this tick species. CLE also play a key role in the reproductive fitness of female *A. americanum* ticks, where clearance of this bacterium by antibiotic treatment reduces egg hatchability, and increases the time to oviposition (Zhong et al., 2007). CLEs have also been identified through field collection in *Rh. turanicus*, where these were higher in females than males, further supporting sex-specific benefits, driven by vertical transmission of these endosymbionts that occurs in *Rh. turanicus* after CLE proliferation and colonization in ovaries (Lalzar et al., 2014). Additional studies have shown that CLE are more abundant in ovaries and Malpighian tubules, but less abundant in other organs as salivary glands (Noda et al., 1997; Lalzar et al., 2014; Wang et al., 2017; Buysse et al., 2019). As *Rh. turanicus* females display higher blood consumption and increased metabolic rates compared with males, sex-specific benefits could be expected to extend beyond reproduction in female ticks of this species, with CLEs suppling essential nutrients in larger quantities in feeding females. Indeed, research has shown a direct positive correlation between the abundance of symbionts and female weight during feeding in this species (Lalzar et al., 2014). In contrast, symbiont levels appeared to be lower in males than in females, where weight increases were also lower during feeding.

In the Cayenne tick, *Amblyomma cajennense*, CLE was identified in salivary glands, ovaries and Malpighian tubules (Machado-Ferreira et al., 2011). Further, this bacterium found with high prevalence rate (100%) in all life stages and in eggs of ticks that confirm its transovarial and transstadial transmission (Machado-Ferreira et al., 2011). Similarly, CLEs proteomes detected from Malpighian tubule (75%) and the ovaries (80%) of the brown dog tick, *Rh. sanguineus* (Cibichakravarthy et al., 2022)possible roles in metabolism, fecundity and osmoregulation (Machado-Ferreira et al., 2011; Sonenshine, 2014; Cibichakravarthy et al., 2022). It is reported that CLEs use few substances from the hemolymph for the synthesis of B vitamins (Magnúsdóttir et al., 2015), potentially explaining why they are rarely found in other tick organs, such as midgut and salivary glands. Nevertheless, the importance of tissue tropism of CLEs has not been fully elucidated (Duron et al., 2017).

*Coxiella*-like endosymbionts have also been found in *Haemaphysalis concinna* collected in different geographical areas of Russia (Mediannikov et al., 2003), and in *Rhipicephalus* in Switzerland (albeit in only a small fraction of the ticks sampled; Bernasconi et al., 2002), Such findings indicate that this symbiont group plays a variable role across different tick species, being important in many of those researched to date. Further investigation of CLEs in ticks could therefore be recommended to explain their role and importance in tick population fitness (Supplementary Table 1).

### Rickettsia

*Rickettsia* is obligate, intracellular gram-negative bacteria (Perlman et al., 2006). These microbes are widely distributed in accordance with their tick hosts across the globe, causing many diseases in humans and animals. Despite being regularly reported as tick symbionts with ticks, however, the symbiotic potential of some species remains poorly understood (Parola and Raoult, 2001; Goddard, 2009). In cases where our understanding is clearer, these symbionts have been shown to play a vital role in tick physiology, fitness and population dynamics and pathogen transmission (Childs and Paddock, 2002). Endosymbiotic *Rickettsia* might have nutritional importance in ticks, as many phylotypes of *Rickettsia* can produce folate (vitamin B), which are not present in the blood of vertebrates on which ticks usually fed (Kagemann and Clay, 2013).

Through various studies, it has been shown that *Dermacentor variabilis* harboring symbiotic *Rickettsia* sp. demonstrate higher motility than ticks without *Rickettsia* sp. (Kagemann and Clay, 2013). *Rickettsia* sp. symbionts are most common in ticks of the genera of *Ixodes*, *Amblyomma*, and *Dermacentor*, whereas it has been less frequently found to in *Rhipicephalus*, *Haemaphysalis*, and *Hyalomma* ticks (Burgdorfer et al., 1973; Nováková and Šmajs, 2018). In *I. scapularis, Rickettsia* endosymbionts has been reported in 100% of eggs, 81% in larva, 90.5% in nymph, and 98.9% in adult females tested (Rounds et al., 2012). Identification of endosymbionts in ticks was done by broad-range polymerase chain reaction and electrospray ionization mass spectrometry. The same study reports this bacterium to be present at high prevalence (100%) in *I. pacificus* (Rounds et al., 2012).

While *Rickettsia* symbionts may play roles in influencing the physiology of ticks, they often found as diverse species assemblages, interacting with one another. *Dermacentor andersoni* harbor symbionts such as *Rickettsia montanensis* (formerly *R. montana*) and *Rickettsia peacockii* (Burgdorfer et al., 1980; Macaluso et al., 2002), for example, and *D. variabilis* can be infected by *Rickettsia rickettsia, Rickettsia bellii*, and *R. montanensis* concurrently (Carmichael and Fuerst, 2006). It has been reported that *R. montanensis* is vertically transmitted in *D. variabilis*, but with this transmission inhibited by co-infection with a second *Rickettsia* sp. (Macaluso et al., 2002). Capillary feeding trials involving PCR diagnostics have also shown that ticks already harboring *Rickettsia* sp. are resistant to challenge with a secondary *Rickettsia* infection. In resisting co-infection, it has been proposed that *Rickettsia* present in tick ovaries change the molecular expression of oocytes, which prevents the occurrence of secondary residential infection (Carmichael and Fuerst, 2006). Soft ticks have also been reported to harbor *Rickettsia*. Particularly, two novel species named *Candidatus* Rickettsia Africa septentrionalis and *Ca.* Rickettsia mauretanica, were detected in *Ornithodoros occidentalis* from Morocco, *Ornithodoros erraticus* from Algeria and *Ornithodoros normandi* from Tunisia (Buysse and Duron, 2020).

### Francisella

*Francisella*-like endosymbionts are gram-negative coccobacilli and facultative intracellular bacteria that are widespread in natural surroundings. In natural ecosystems, the survival of *Francisella* depends on temperature, direct sunlight exposure, and other physical factors (Kılıç, 2010; Celli and Zahrt, 2013). These bacteria are probably sustained in the environment through associations with various animals, including lagomorphs, rodents, insectivores, carnivores, ungulates, marsupials, birds, amphibians, and various species of invertebrates. Transmission to/between animals and humans may occur *via* bites of both ticks and mosquitoes (Momer, 1992; Eliasson et al., 2006). Ticks may serve as reservoirs, carrying the bacteria in their bodies throughout their lives, including in their salivary glands from where *Francisella* may be transmitted to new hosts at the site of tick feeding (Goethert and Telford, 2005; Gürcan, 2014; Yeni et al., 2021). *Francisella* have also been found in the reproductive tissues of female *D. andersoni* (Niebylski et al., 1997), with instances of unfed, infected larvae of *A. americanum* occurring in nature. Supporting the idea that transovarial transmission can occur (Calhoun and Alford, 1955). Transstadial transmission of *Francisella tularensis* has also been confirmed under laboratory conditions for multiple tick species, including *D. andersoni*, *D. variabilis* and *A. americanum* (Petersen et al., 2009).

FLEs are assumed to be non-pathogenic to humans, though they may cause limited pathogenicity in small animals (Keim et al., 2007) and are found in human-biting ticks, including those belonging to the genera *Dermacentor*, *Amblyomma*, *Ixodes*, and *Hyalomma* (Scoles, 2004; Machado-Ferreira et al., 2009; Ivanov et al., 2011; de Carvalho et al., 2016; Azagi et al., 2017). Presence of the genus *Francisella* has been reported in the camel tick, *Hyalomma dromedarii*, through 16s rRNA sequencing, with high relative abundance (99%) (Perveen et al., 2020b) and subsequent PCR confirming close relation to FLE (Perveen et al., 2021a). In the same geographic area, *H. dromedarii* ticks were reported throughout the year with high prevalence (94%) (Perveen et al., 2020a). It is possible that the high prevalence of camel ticks reported in this work may have been due to high abundance of these endosymbionts, though favorable microclimatic/environmental conditions and abundance of hosts for blood feeding (due to an increase in camel farming) may have also played a role. Previously, FLE was also detected with high prevalence in *Hyalomma* species (90.6%). In addition, maternal transmission rates of up to 91.8% were reported and FLE were localized in Malpighian tubules, ovaries, and salivary glands in *H. marginatum* (Azagi et al., 2017).

*Francisella*-like endosymbionts are closely related to pathogenic species of the genus *Francisella* (Noda et al., 1997; Scoles, 2004). Therefore, precise identification of this endosymbiont, its prevalence and interactions with other members of tick microbiota (co-existence) are crucial to understand and estimate its pathogenic potential, and investigate possible transmission to humans and animals. For example, the presence of closely related FLEs in tick species, including *D. variabilis*, *D*. *andersoni*, and *D. occidentalis* (Staples et al., 2006; Petersen et al., 2009) that can sustain and transmit pathogens causing tularemia poses a public health challenge.

*Francisella*-like endosymbiont is needed for tick growth and life cycle (Duron et al., 2018). In addition, FLEs have been found to be dominant symbionts in the Gulf Coast tick, *Amblyomma maculatum* microbiome (Binetruy et al., 2020) and reported in soft as well as hard ticks, especially in species belonging to the *Ornithodoros* genera (Gerhart et al., 2018). FLEs affect tick nutrition, and their capability to synthesize vitamin B make them especially suitable as mutualistic symbionts with *Ornithodoros moubata* (Duron et al., 2018). Further, elimination of FLE cause physical abnormalities and deficiencies in ticks that were restored by providing supplement of B vitamins (Duron et al., 2018). Gerhart et al. (2016) demonstrated the metabolic capability of FLE of *A. americanum* and found that FLE synthesizes cysteine, threonine, tyrosine, tryptophan, phenylalanine, and serine from pyruvate, and can break down glutamate, glutamine, and asparagine into ATP. This higher biosynthetic capability of FLE-Am could have led to replacing an ancestral symbiont, CLE in *A. maculatum* (Gerhart et al., 2016). Therefore, FLEs improve tick fitness by supplying vitamins and cofactors found in low concentrations in vertebrate blood (Gerhart et al., 2018).

### Wolbachia

*Wolbachia* is an intracellular gram-negative bacterium that is the most commonly found microorganism infecting various arthropods. This bacterium is present in almost 60% of insect species and plays a role in multiple mechanisms, including altering host reproduction by inducing reproductive disorders, driving parthenogenesis, and acting as a defensive endosymbiont (Sazama et al., 2019). The presence of *Wolbachia* in ticks has been associated with parasitism by an *Ixodiphagus* parasitoid (Plantard et al., 2012; Luu et al., 2021). *Ixodiphagus hookeri* (Hymenoptera, Chalcidoidea, and Encyrtidae) that are endoparasitoids of *I. ricinus*, were found infected with *Wolbachia pipientis* (99.2% prevalence) (Plantard et al., 2012). Further, it was reported that natural populations of *I. ricinus* ticks harboring *Wolbachia* were parasitized *by I. hookeri*, and ticks that were not parasitized by *I. hookeri* were *Wolbachia*-free (Plantard et al., 2012). Therefore, the occurrence of *W. pipientis* in *I. ricinus* ticks is probably attributable to the presence of *I. hookeri* (Plantard et al., 2012). Excluding the transmission of *Wolbachia* from *Ixodiphagus* to ticks, evidence suggests that *Wolbachia* is rarely found in ticks and other maternally inherited endosymbionts as *Spiroplasma*, *Midchloria*, or *Rickettsiella* are more common (Tijsse-Klasen et al., 2011). In agreement with this, this bacterium has been reported with a prevalence rate of only 14% in *I. ricinus* (Subramanian et al., 2012). Likewise, in *A. americanum* it was found with a prevalence rate of 3.5–25% in females (Zhang et al., 2011).

In a study in Southern Maryland (United States), *Wolbachia* infection was present only at low frequency in nymphs of *A. americanum*, and observed primarily in females and only rarely in males. This significant difference in male and female ticks could be due to male-killing effects of infection or a sampling bias. The impact of *Wolbachia* species on the reproductive potential of *A. americanum* is still unknown and warrants further attention to investigate any relationship (Benson et al., 2004). Furthermore, a more extensive range of tick species should be examined for *Wolbachia*, evaluating sex-specific infection rates and the role of this bacterium in host reproduction processes. Indeed, this bacterium may have a potential role in the biological control of ticks, as it does for various disease vectors and other arthropod pests (Ahantarig and Kittayapong, 2011).

### *Candidatus* Midichloria Mitochondrii

*Candidatus* Midichloria mitochondrii is an intracellular bacterium belonging to the order Rickettsiales (Stavru et al., 2020), mostly reported in *I. ricinus* and this bacterium has vertical transmission in this tick (Sassera et al., 2006; Mariconti et al., 2012a). This alphaproteobacterium colonize mitochondria and was found residing in the mitochondrial intermembrane space through electron microscopy (Sassera et al., 2006; Stavru et al., 2020). It has been noted as the most prevalent endosymbionts in *I. ricinus*, having 100% (Olivieri et al., 2019), and 44% (Sassera et al., 2006) prevalence rates in females and males, respectively. *Candidatus* Midichloria mitochondrii was found to be abundant in ovaries of female *I. ricinus* (Olivieri et al., 2019) and vertically transmitted symbiont reported with 100% prevalence in females and immatures. Further, *Ca.* Midichloria mitochondrii can be found in gut, rostrum, tracheae, Malpighian tubules, and salivary glands of *I. ricinus* females (Olivieri et al., 2019). Results suggest subpopulations of *Ca.* Midichloria mitochondrii with different specializations due to tissue tropism. Further, *in silico* metabolic reconstruction indicate that *Ca.* Midichloria mitochondrii could enhance the host fitness, help in the anti-oxidative defense, maintain of homeostasis, water balance, and stable its population through vertical and horizontal transmission in the tick host (Olivieri et al., 2019). *In silico* metabolic reconstruction from the *Ca.* Midichloria mitochondrii genome showed that several genes involved in interaction with *I. ricinus* and complete biosynthesis pathways for B vitamins, especially B9 (folate) and B7 (biotin) suggest its nutrient-provisioning role. However, more experiments are required to better understand the mechanisms underlying this symbiotic interaction.

Mariconti et al. (2012a) and Perlman et al. (2006) showed that 47 patients exposed to bites of *Ixodes ricinus* ticks were seropositive to antigens of *Ca.* Midichloria mitochondrii. This suggests that *Ca.* Midichloria mitochondrii antigens secreted together with tick saliva could trigger the human immune response, or that ticks could transmit this endosymbiont to humans. The second scenario raises the possibility that *Ca.* Midichloria mitochondrii can then be acquired by a second tick feeding on the same host. Making this an example of an endosymbiont completing a tick-to-host-to-tick transmission route similar to that reported of tick-borne pathogens. Whether, in addition to the vertical transmission, such host-mediated transmission route plays any role on *Ca.* Midichloria mitochondrii life cycle remains unknown. Coincidentally, patients found seropositive with *Ca.* Midichloria mitochondrii were among 80 tick-exposed patients hospitalized due to clinical signs of Lyme disease, and 31 of them were also found seropositive for *B. burgdorferi* sensu lato (Mariconti et al., 2012a). Deducing a direct relation between *B. burgdorferi* and *Ca.* Midichloria mitochondrii colonization in *I. ricinus* from co-occurrence data in ticks is not justified, as this endosymbiont is present in nearly 100% of tick females, while *Borrelia* prevalence in ticks is highly variable. However, whether patient exposure to *Ca.* Midichloria mitochondrii favors, or not, *Borrelia* infection and Lyme disease is an interesting, but unanswered, question. A previous report reported that the growth of a pathogenic *R. parkeri* in *A. maculatum* along with endosymbionts (Budachetri et al., 2018). Further, Budachetri et al. (2018) found that *R. parkeri* interaction with tick symbionts, FLE and *Ca.* Midichloria mitochondrii (CMM) modulate host’s defenses by up-regulating tick selenoproteins.

## Mutualistic “Pathogens”

TBPs considered as having a “mutualist” relation with the tick, but not symbiotic. Some TBPs have vertical and horizontal transmission (e.g., *Babesia*) (Young et al., 2019) and others only horizontal (*A. phagocytophilum* and *B. burgdorferi*) (Jaarsma et al., 2019; Kurokawa et al., 2020). During blood feeding, various microbes are acquired that can trigger an immunological response in ticks. The immune system of ticks includes various enzymes and proteins, with endosymbionts also known to interact with tick immune function. For instance, *B. burgdorferi* can be phagocytosed by tick hemocytes (Zchori-Fein and Bourtzis, 2011; Hussain et al., 2021a). For instance, *l. scapularis* transmits the Lyme disease spirochete, *B*. *burgdorferi* whereas the *D. variabilis* is unable to transmit the bacterium to vertebrate host (Johns et al., 2001). It’s due to immune function of both tick species. In *I. scapularis*, some *Borrelia* spirochete were found connected with hemocytes, while several spiral-shaped intact bacteria were free in the hemolymph, however, in *D. variabilis* intact spirochetes were very less. Further, *I. scapularis* tissues contained culturable bacteria unlike *D. variabilis* tissues (Johns et al., 2001). *In vitro*, it was demonstrated that spirochetes motility and survival not reduced when they were incubated with *I. scapularis* hemolymph plasma, however, more than 50% spirochetes became non-motile after incubation of spirochetes with *D. variabilis* hemolymph plasma. Furthermore, *I. scapularis* showed immunotolerance against *B. burgdorferi* and slow phagocytic response whereas, *D. variabilis* showed high immunocompetence and rapid phagocytic activity to clear pathogens (Johns et al., 2001). *A. phagocytophilum*, an intracellular bacteria transmitted by *I. scapularis.* Its transmission is restricted by a member of the 5.3 kDa family of antimicrobial peptides expressed in the salivary glands of tick (Liu et al., 2012). Studies have been conducted on *A. phagocytophilum* how it manipulate gene expression and activate signaling pathways (Cabezas-Cruz et al., 2017). For instance, infection of *A. phagocytophilum* stimulates the expression of antimicrobial peptides that is mediated by the activation of the Janus kinase-signal transducer and activator of transcription (JAK-STAT) pathway (Liu et al., 2012) to control bacterial load in tick salivary glands. Cabezas-Cruz et al. (2017) discussed about how tick-pathogen interactions increase the fitness in tick hosts. Pathogens manipulate tick protective responses to facilitate infection, however, preserve tick feeding and vector capacity to assure the survival of the pathogens as well as ticks (Cabezas-Cruz et al., 2017). *A. phagocytophilum* infections in *I. scapularis* showed higher tick fitness due to the induced expression of a tick antifreeze glycoprotein that increases their survival in the cold conditions (Neelakanta et al., 2010).

## Symbiont-Pathogen Interactions

Ecological relationships among organisms are broad and diverse. The interactions that underpin them can range from beneficial to harmful along a continuum (Pérez-Brocal et al., 2011). Accurately defining symbiotic, mutualistic, or pathogenic interactions is challenging, especially where they may be influenced by various external factors such as climate change, land-use change, and invasion of parasites and hosts into new geographic ranges. Modern methodologies are helping researchers to better map this complex landscape of interactions, where molecular-based diagnostics have proven especially useful in confirming associations between microbes and various protozoa, other bacteria, fungi, and/or animals (Pérez-Brocal et al., 2011).

Multiple pathogens (Baneth, 2014) and symbiotic bacteria (Noda et al., 1997) may co-exist in tick midgut. But, many endosymbiotic microorganisms have established themselves in the ovaries of female ticks (Noda et al., 1997), and are often transmitted to eggs (and subsequently nymphs) from the mother. Examples can be found from *Coxiella*-, *Francisella-*, and *Rickettsia*-like endosymbionts, which all affect/improve the host fitness (Ahantarig et al., 2013), and defense against environmental stress (Bonnet et al., 2017). In some cases, these endosymbionts may infect humans (Ahantarig et al., 2013), with this potentially then affecting the tick-borne microbes occurrence and transmission (Bonnet et al., 2017; Bonnet and Pollet, 2021). Pre-existence of another symbiont can also influence the performance of a different species. For instance, the vertical transmission of a second *Rickettsia* species in the *D. variabilis* is inhibited by pre-existing Rickettsial infection in these ticks (De Sousa et al., 2001). For example, *R. peacockii* is found in Rocky Mountain wood ticks, *D. andersoni* and displays a close phylogenetic relationship to *Rickettsia rickettsii*, a bacteria of the spotted fever group (SFG), but many of the characteristics of rickettsial pathogens are missing (Burgdorfer et al., 1980). The major difference between pathogenic and non-pathogenic SFG *Rickettsia* is the ability to proliferate within macrophages distinguishable factor in pathogenic *Rickettsia*. In addition, pathogenic *Rickettsia* may be able to increase the endoplasmic reticulum protein folding capacity, while non-pathogenic *Rickettsia* do not show such capacity (Curto et al., 2019). Similarly, *-*FLEs- have been detected in a range of tick genera, such as *Amblyomma, Hyalomma, Ixodes*, and *Ornithodoros* (Sun et al., 2000; Scoles, 2004; Machado-Ferreira et al., 2009; Ivanov et al., 2011; de Carvalho et al., 2016; Azagi et al., 2017). The occurrence of closely related *Francisella* sp. in *Dermacentor.* species that can transmit tularemia (Elston and Apperson, 1977) suggests an important role for precise screening of *F. tularensis* in laboratories by PCR (Kugeler et al., 2005). Indeed, full assessment of the pathogenic potential of FLEs is crucial and has recently been undertaken for *Rickettsia* species (Felsheim et al., 2009). This work demonstrated that symbionts previously considered to be non-pathogenic (Raoult and Roux, 1997) may in fact be the opposite, as was true for *Rickettsia slovaca* and *Rickettsia helvetica* (Raoult and Roux, 1997). This supports that the presence, infection rate, and ecological/biological roles of bacterial endosymbionts need to be examined closely to reveal their contribution to tick-borne diseases epidemiology.

## Impact of Symbionts on Disease Ecology

Vector-borne zoonotic diseases are a global health threat and involve humans, pathogens, vectors, and wildlife (Harrus and Baneth, 2005; Collinge and Ray, 2006). Novel disease emergence can be especially significant and may be facilitated through the anthropogenic spread of disease vectors and pathogens across the globe. Virulent lineages of pathogens can also be distributed globally through anthropogenic activities, such as the international trade of animals (Cunningham et al., 2017), deforestation, agricultural development, and the magnitude of human interaction in the disease ecosystem, with climate change and socio-economic and environmental factors also influencing the dynamics of pathogen populations (Paul et al., 2016). The regional pattern of risk of pathogen transmission and circulation in an area is very important to human health and influenced by several factors. Spatial distribution of tick-borne pathogens, microclimatic conditions, and host abundance may affect pathogen dynamics, as well as the survival of ticks as potential vectors. Host species presence and abundance may impact pathogen spread (Estrada-Peña and de la Fuente, 2014), as may host and tick vector competence, as influenced by vector density and longevity (de la Fuente et al., 2017). Logically, any habitat considered fit for the tick-borne pathogen’s circulation and spread must meet the prerequisite of housing ticks and their hosts for any risk to be realized (Pfäffle et al., 2013). High-throughput sequencing approaches have also emphasized the potential implications of the composition, diversity, and functional role of tick microbial fauna to public health (de la Fuente et al., 2017).

Symbionts may impact disease ecology in two ways. Firstly, through their impact on tick physiology they can contribute to increase fitness and vector abundance which indirectly increases pathogen circulation and disease risk. Secondly, symbionts compete or favor pathogen colonization and can directly impact disease ecology by decreasing or increasing the chance of host being bitten by tick able to transmit pathogens.

Symbionts may benefit ticks through impacts on their survival, growth, and defense systems (Ahantarig et al., 2013; Bonnet and Pollet, 2021), all of which can have knock-on effects to their significance as disease vectors for humans. The assessment of symbiont associations is very important because these impact tick reproduction and fitness (Ahantarig et al., 2013). For example, a decrease in progeny number and increase in oviposition time has been reported in *A. americanum* following the elimination of CLEs through antibiotics (Zhong et al., 2007). Other key processes in ticks, as supported through their symbionts, could potentially be targeted in a similar fashion. *Candidatus* Midichloria mitochondrii in *I. ricinus* affect tick molting process (Zchori-Fein and Bourtzis, 2011; de la Fuente et al., 2017). Further, recent sequencing and analysis of this bacterium genome suggest that the bacteria may serve as a source of ATP for the host cell during oogenesis (Epis et al., 2008; Gnainsky et al., 2021). *Rickettsia*-infected *Dermacentor* ticks showed more motility than uninfected ticks (Kagemann and Clay, 2013). Higher motility associated to host-seeking behavior in ticks indirectly influences infection risk by increasing the rates of tick bites and pathogen transmission. Therefore, for a given tick species, various symbionts and their population rate that varies across geographic locations (Lalzar et al., 2012; Duron et al., 2017) affect vector fitness, their abundance and impact disease risks.

Symbionts may interfere with pathogens replication and transmission by affecting pathogen diversity and abundance in various tick species and their transmission to humans and animals (Bonnet et al., 2017). Because symbionts compete with pathogens for nutrients or tissues and may excrete molecules directly inhibiting the growth of pathogens or facilitate pathogens development by immunosuppressing the invertebrate hosts (Bonnet et al., 2017). For example, *Wolbachia* may affect with the replication and transmission of pathogens such as bacteria, viruses, protozoa, etc. and protect arthropods from parasite-induced mortality, may be through up-regulating the immune system (Brownlie and Johnson, 2009). Further, *Wolbachia* can cause pathogen interference by reducing the chance of pathogen infection and decrease pathogen load, and cytoplasmic incompatibility by reducing hatchability of eggs (when infected males mate with uninfected females) in mosquitoes (Caragata et al., 2016). Several pathogens may affect vector competence (Hajdusek et al., 2013). Tick innate immunity involves several cellular and humoral response pathways that mediate defense to various infections caused by microorganisms, such as *Borrellia, Flavivirus*, and Babesia (Hajdusek et al., 2013; de la Fuente et al., 2017). Tick symbionts may influence tick immunity that in turn may influence pathogen infection. FLE in *D. andersoni* was found positively associated with pathogenic *Francisella novicida* infection (Gall et al., 2016). The presence of FLEs has positively influenced the establishment of *F. novicida* in *D. andersoni* that may suppress the tick immune system favoring the acquisition of *F. novicida* (Gall et al., 2016). Therefore, these microbial associations allow symbionts to facilitate or limit pathogen transmission and directly influence vector-borne infections.

## Using Obligate Hematophagy and Endosymbionts as Weak Spots for the Control of Ticks and Tick-Borne Pathogens

As obligate hematophagous parasites, ticks ingest large amounts of blood from the vertebrate host during feeding. The tick midgut is the first organ in contact with host immune components present in the blood. After crossing the gut barrier (Ackerman et al., 1981; Ben-Yakir et al., 1987; Wang and Nuttall, 1994), host antibodies (Willadsen, 1997) and complement proteins (Rathinavelu et al., 2003) can reach the tick hemolymph (Ackerman et al., 1981; Ben-Yakir et al., 1987; Wang and Nuttall, 1994) and access the tick ovaries and eggs (Galay et al., 2018) as well as salivary glands and be secreted back to the host (Wang and Nuttall, 1994). For example, in *D. variabilis* and *I. scapularis* ticks, the crossing of host IgG from the midgut into the hemocoel occur during the later phases of engorgement (Vaughan et al., 2002). Notably, the immune functions of antibodies and complement are retained in the tick tissues (Ackerman et al., 1981; Ben-Yakir et al., 1987; Wang and Nuttall, 1994). Host antibodies interact not only with tissues and surface proteins (Willadsen, 1997), but can also be specifically transported inside the tick cells where they can interact with intracellular proteins (de la Fuente et al., 2011; Rodríguez-Mallon et al., 2012, 2015). Functional host antibodies have also been shown to interact with symbionts in *Rodnius prolixus* (Ben-Yakir, 1987), and *Glossina morsitans* (Nogge, 1978), as well as with bacterial microbiota in mosquitoes (Noden et al., 2011) and ticks (Mateos-Hernández et al., 2020, 2021).

Targeting vector microbiota with host antibodies is the rationale behind anti-microbiota vaccines for the control of vector arthropods such as ticks (Mateos-Hernández et al., 2020, 2021). Immunization with a tick microbiota Enterobacteriaceae, caused significant mortality of engorging ticks (Mateos-Hernández et al., 2020). Antibodies against the glycan α-Gal, present in tick bacterial microbiota, were associated with a mean mortality of approximately 45% in ticks fed on α1,3GT-deficient mice (Mateos-Hernández et al., 2020). Anti-microbiota vaccine directed at Enterobacteriaceae in the microbiota of *I. scapularis* disrupted both the makeup and functions of the microbiome and decreased pathways central to lysine degradation (Mateos-Hernandez et al., 2021). Anti-microbiota vaccines are a microbiome manipulation tool for the induction of infection-refractory states in the vector microbiome (Maitre et al., 2022).

The preliminary results with anti-microbiota vaccines justify their use to target tick endosymbionts for vector and/or tick-borne pathogen control. *Rhodnius prolixus* fed exclusively on blood from rabbits immunized against the symbiont *Rhodoccocus rhodnii* have developmental alterations such as prolonged molting times, incomplete development, and malformed limbs (Ben-Yakir, 1987). Feeding of *Rhodnius prolixus* larvae on hosts immunized against their symbiont produces retardation of the symbiont growth (Ben-Yakir, 1987). Developmental alterations observed in *R. prolixus* fed on *Rhodococcus rhodnii*-immunized animals were similar to those described in aposymbiotic triatomines (sterile raised and germ-free insects that lack *R. rhodnii*) (Salcedo-Porras et al., 2020). Similar results were obtained by Nogge (1978) who found that tsetse flies fed on rabbits immunized with symbionts became aposymbiotic and their fecundity decreased drastically while their longevity was not affected. Furthermore, *Glossina morsitans morsitans* flies maintained on rabbits immunized with gut bacteria had high mortality rates and permanently laterally extended wings, which in turn impairs flying and therefore trypanosomes transmission (Kaaya and Alemu, 1984). In addition to targeting tick endosymbionts with live-bacteria vaccines (Mateos-Hernández et al., 2020, 2021), selected enzymes encoded in the symbiont genome and transcriptionally active could also be targeted with host antibodies.

## Conclusion and Perspectives

In this review, we have discussed five main tick-borne endosymbiont groups, comprising *Coxiella-, Rickettsia-*, *Francisella*-, *Wolbachia*, and *Midichloria -*like species, reporting key symbiont-pathogen interactions and exploring potential impacts of symbionts and their associations on tick physiology and tick-borne disease ecology. Examples are provided of various bacteria that can benefit their host’s health, with further examples of opportunistic or pathogenic bacteria that may stress the host in some way while exploiting it. It is clear from many of these examples that tick-symbiont relationships are complex and context-specific, and better defining the pathways that dictate how pathogenic microbes contribute to disease ecology will allow improved treatments that target the virulence responses associated with this microbiota.

Endosymbionts play a significant role in providing nutrients (Greay et al., 2018) that support host persistence, for example, rickettsial symbionts of *D. andersoni* and *Amblyomma americanum*, and the CLE of *A. Americanum*, and are essential for the fitness of their tick hosts (Greay et al., 2018). Experimentation using antibiotics have shown that cleansing ticks from its bacterial endosymbionts have major negative impact of vector fitness and survival as well as vector competence. For example, *D. andersoni* exposure to oxytetracycline caused a significant decline in tick feeding, molting competence, and survival (Wade et al., 2014). Similarly, injecting laboratory-reared *A. americanum* ticks with rifampin resulted in reduced endosymbionts (*Coxiella* sp. and *Rickettsia* sp.) and a reduction in weight of adult ticks post-molting (Zhong et al., 2007). However, antibiotic overuse is associated to several undesirable effects such as the emergence of microbial antibiotic resistance, agro-ecosystem contamination, and alteration on animal and human microbiota with potential negative effect on health. In addition, the effect of antibiotics on the microbiota is not specific, as several bacterial species can be depleted by antimicrobial treatment. All this, had led to the implementation of strong regulations to limit antibiotic use, particularly in livestock. Therefore, preventing the use of antibiotics to target tick endosymbionts. In this context, anti-microbiota vaccines emerge as an environmentally friendly alternative to target endosymbionts for the control of ticks and tick-borne pathogens.

The latest high throughput molecular diagnostic approaches have enabled researchers to explore microbiomes of different arthropods species rapidly and cost-effectively. However, the microbial composition of many tick species still needs to be explored, and additional studies are required to understand the functional role of these microorganisms in disease ecology and epidemiology (Greay et al., 2018). Tick-borne infections are a severe threat to public health and food security globally, and researchers, scientists, public health professionals and veterinarians must work both independently and collaboratively to reduce the risk they pose. Vector-borne infections thrive where infectious pathogens, competent vectors, and an infection-compatible microbiome exist, with disruption of any one of these drivers likely to reduce overall pathogen transmission by vectors (Maitre et al., 2022). Manipulation and targeting of tick endosymbionts have been under-explored to this end to date but may provide significant opportunities in the future.

## Author Contributions

SH conceived the study and searched the literature. SH and OS planned and designed the review manuscript. SH, NP, AC-C, and OS screened and organized the data. SH, NP, AH, AC-C, and OS drafted the manuscript. AC-C provided intellectual inputs and share ideas. SH, NP, AH, AC-C, OS, MA, BS, and JZ revised the manuscript. OS, AC-C, DG, and JL critically revised the manuscript. All authors read and approved the final manuscript.

## Conflict of Interest

The authors declare that the research was conducted in the absence of any commercial or financial relationships that could be construed as a potential conflict of interest.

## Publisher’s Note

All claims expressed in this article are solely those of the authors and do not necessarily represent those of their affiliated organizations, or those of the publisher, the editors and the reviewers. Any product that may be evaluated in this article, or claim that may be made by its manufacturer, is not guaranteed or endorsed by the publisher.
