# Supplementary material for: The Symbiotic Continuum Within Ticks: Opportunities for Disease Control
Source: Front Microbiol. 2022 Mar 17;13:854803. doi: 10.3389/fmicb.2022.854803 (PMC8969565; doi:10.3389/fmicb.2022.854803)
Supplement: Supplementary file 1 [file Table_1.doc]

| **Bacterial genera (endosymbionts)** | **Tick genera/species** | **Host** | **Country (Tick sampling site)** | **Detection method** | **Presence (%)** | **References** |  |
| --- | --- | --- | --- | --- | --- | --- | --- |
| Table 1. Some illustrative examples -of endosymbionts, their distribution and prevalence in different tick species. | | | | | | |  |
| ***Candidatus* Midichloria mitochondrii** | | | | | | |  |
|  | *Ixodes ricinus* | Hamster | Germany | PCR | Female: 100 %,  Male: 44 % | (Sassera, Beninati et al. 2006) |  |
|  | *I. ricinus* | Field-collected | Czech Republic | PCR |  | (Sassera, Lo et al. 2008) |  |
|  | Ixodid ticks |  | Italy, North America and Iceland | PCR |  | (Epis, Sassera et al. 2008) |  |
|  | *Ixodes holocyclus* | Domesticated and wild animals, vegetation | Australia | PCR |  | (Beninati, Riegler et al. 2009) |  |
|  | *Rhipicephalus sanguineus, Hyalomma*spp. | Field-collected | Israel | PCR | *Rh. sanguineus*: 4.2%,  *Hyalomma*: 38.5% | (Harrus, Perlman-Avrahami et al. 2011) |  |
|  | *Dermacentor andersoni* | Field-collected | Canada | PCR |  | (Dergousoff and Chilton 2011) |  |
|  | *I. ricinus* | Goats and hedgehogs | Italy | PCR |  | (Mariconti, Epis et al. 2012) |  |
|  | *Rhipicephalus (Boophilus) decoloratus, I. ricinus* |  |  | PCR |  | (Najm, Silaghi et al. 2012) |  |
|  | *Amblyomma americanum* | Vegetation | USA | 16S rRNA gene pyrosequencing |  | (Williams-Newkirk, Rowe et al. 2012) |  |
|  | *I. ricinus* | Goats and dogs, vegetation | Italy | PCR, Fluorescence in situ hybridization (FISH) |  | (Epis, Mandrioli et al. 2013) |  |
|  | *I. holocyclus* | Mammals and field-collected | Australia and Germany | PCR |  | (Gofton, Oskam et al. 2015) |  |
|  | Ixodidae family and Argasidae family | Animals, field collected |  | PCR |  | (Gofton, Oskam et al. 2015) |  |
|  | *Amblyomma maculatum* | Laboratory-reared | USA | qRT-PCR analysis |  | (Budachetri, Kumar et al. 2018) |  |
|  | *Rh. sanguineus s.l.* | Cat | Thailand | PCR |  | (Trinachartvanit, Rakthong et al. 2018) |  |
|  | *I. ricinus* | Killed wild roe deer | Italy | Quantitative PCR | 100% | (Olivieri, Epis et al. 2019) |  |
|  | *I. ricinus* | Freshly-culled fallow deer | Scotland | Quantitative PCR |  | (Al-Khafaji, Clegg et al. 2019) |  |
| ***Coxiella*-Like Endosymbiont (CLE)** | | | | | | |  |
|  | *Haemaphysalis concinnae* | Wild vegetation | Russia | PCR |  | (Mediannikov, Ivanov et al. 2003) |  |
|  |  |  |  |  |  |  |  |
|  | *A. americanum* | Laboratory-reared and field-collected | USA | 16S rRNA gene sequencing and diagnostic PCR |  | (Kobayashi, Chatanga et al. 2021) |  |
|  | *A. americanum* | Laboratory-reared and field-collected | USA | Quantitative PCR |  | (Jourdain, Duron et al. 2015) |  |
|  | *Amblyomma cajennense* | Field-collected | Brazil | PCR-RFLP Detection  Real-Time qPCR Assay  Fluorescent In Situ Hybridization | 100% | (Machado-Ferreira, Piesman et al. 2009) |  |
|  | *Rh sanguineus, Rhipicephalus turanicus* | Field-collected | Israel | 16S rRNA gene fragments |  | (Lalzar, Harrus et al. 2012) |  |
|  | *A. americanum*  *Rh sanguineus* | Laboratory-reared and field-collected | USA | Broad-Range PCR/ESI-MS | 75%  100% | (Rounds, Crowder et al. 2012) |  |
|  | *Dermacentor silvarum* | Field-collected | China | Diagnostic PCR assays | 95.6% | (Liu, Li et al. 2016) |  |
|  | *A. americanum* | Laboratory-reared | USA | V3-V4, 16S rDNA, Illumina MiSeq,  Genome Sequencing  Illumina HiSeq 2000 | 95% | (Smith, Driscoll et al. 2015) |  |
|  | *Rhturanicus* | Field-collected | Israel | qPCR analysis  Genome analysis |  | (Gottlieb, Lalzar et al. 2015) |  |
|  | *Haemaphysalis hystricis,*  *Haemaphysalis lagrangei,*  *Haemaphysalis obesa,*  *Haemaphysalis shimoga* | Field-collected | Thailand | PCR, Sanger sequencing | 59% in female ticks | (Arthan, Sumrandee et al. 2015) |  |
|  | *Rhipicephalus bursa* | Field-collected | Italy | LAMP assay  PCR, Sanger sequencing |  | (Rahal, Medkour et al. 2020) |  |
|  | *Ornithodoros capensis*  *Ornithodoros peruvianus*  *Rhipicephalus annulatus*  *Rh. decoloratus*  *Rhipicephalus geigyi*  *Ornithodoros sonrai* |  |  | Quantitative PCR |  |  |  |
|  | *Argas monachus*  *Ornithodoros amblus*  *Ornithodoros brasiliensis*  *O.capensis*  *Ornithodoros maritimus*  *Ornithodoros peruvianus*  *Ornithodoros spheniscus*  *Haemaphysalis punctata*  *Rh. decoloratus*  *Rhipicephalus* sp.1and sp.2 |  | Argentina  Peru  Brazil  Mozambic  Tunisia  Chile  Chile  England  Burkina-Faso  Ivory Coast | Nested PCR | 29.7% | (Duron 2015) |  |
|  | *Ornithodoros muesebecki* | Socotra Cormorant colony | UAE | [Multiplex PCR](https://www.sciencedirect.com/topics/immunology-and-microbiology/multiplex-polymerase-chain-reaction), Sanger sequencing |  | (Al-Deeb, Frangoulidis et al. 2016) |  |
|  | *Amblyomma coelebs, Amblyomma calcaratum, Amblyomma oblongoguttatum, Amblyomma variegatum, Amblyomma gemma, Amblyomma lepidum, Amblyomma javananse, Amblyomma nodosum, Amblyomma dubitatum, Amblyomma rotundatum,*  *Rh. sanguineus s.l.*  *Rhipicephalus microplus* | Horses, mules, domestic dogs, anteaters, cattle, armadillos, and humans | Brazil | PCR detection | 100%  37% | (Machado-Ferreira, Vizzoni et al. 2016) |  |
|  | *A. maculatum* | Laboratory-reared | USA | Genome Sequencing,  Illumina Hi-Seq 2500 |  | (Gerhart, Moses et al. 2016) |  |
|  | *Haemaphysalis bispinosa,*  *Dermacentor compactus,* and *Amblyomma*sp. | Wild boars, porcupine | Malaysia | PCR, nested-PCR |  | (Khoo, Lim et al. 2016) |  |
|  | *Haemaphysalis longicornis* | Horses | South Korea | Nested PCR and 16S rRNA sequencing | 52.4% | (Seo, Lee et al. 2016) |  |
|  | *Rh. microplus* | Cattle | Brazil | PCR | 98% | (Guizzo, Parizi et al. 2017) |  |
|  | *Rhipicephalus, Argas, Ornithodoros, Amblyomma Haemaphysalis, Dermacentor, Hyalomma,* and *Ixodes.* | Laboratory-reared and field-collected |  | PCR, Sanger sequencing | 60.5% | (Bonnet, Binetruy et al. 2017) |  |
|  | *Ha. longicornis* |  | China | Semi-quantitative PCR |  | (Zhang, Norris et al. 2011) |  |
|  | *Dermacentor marginatus* *Rhipicephalus bursa, Rh. sanguineus s.l.* | Goats | Greece | PCR, Sanger sequencing |  | (Papa, Tsioka et al. 2017) |  |
|  | *Haemaphysalis tibetensis* | Vegetations | China | PCR, RT-qPCR | 100% | (Wang, Li et al. 2017) |  |
|  | *Haemaphysalis wellingtoni,  Haemaphysalis obesa,  Haemaphysalis bispinosa, Amblyomma testudinarium* | *Gallus gallus domesticus*, *Gallus gallus*, *Lophura diardi* | Thailand | PCR, Sanger sequencing |  | (Trinachartvanit, Wutha et al. 2019) |  |
|  | *Rh. microplus* | Laboratory-reared | Texas | Genome Sequencing,  Illumina Hi-Seq 2500 |  | (Ramaiah and Dasch 2018) |  |
|  | *I. ricinus, Dermacentor reticulatus* | Field-collected | Slovakia | PCR-based methods | 32.7% | (Špitalská, Sparagano et al. 2018) |  |
|  | *Ornithodoros maritimus, D. marginatus,* and *Ixodes hexagonus,* | Field-collected, seabirds, horses, hedgehogs | France | PCR, Sanger sequencing  Fluorescence in situ hybridization |  | (Buysse, Plantard et al. 2019) |  |
|  | *Dermacentor everestianus* | Sheep, yaks and horses. | China | PCR | 100% | (Li, Li et al. 2019) |  |
|  | *Ha. bispinosa* | Goat | Thailand | PCR |  | (Takhampunya, Sakolvaree et al. 2021) |  |
|  | *A. americanum* | Vegetation | Georgia | Ion Torrent PGM  qPCR assays |  | (Dasch, Ramaiah et al. 2019) |  |
|  | *Rh. sanguineus* | Field-collected | Israel | V4 region, 16S rRNA gene  Illumina MiSeq platform  Quantitative PCR | 99.69 female field collected ticks | (Ben-Yosef, Rot et al. 2020) |  |
|  | *D. marginatus,*  *Haemaphysalis sulcata,*  *Hyalomma excavatum,*  *Hyalomma detritum, Hyalomma lusitanicum, Hyalomma marginatum*  *Rh. bursa,*  *Rh. sanguineus s.l.*  *I. ricinus* | Dairy cattle | Algeria | qPCR | 51.7 % | (Raele, Galante et al. 2015) |  |
|  | *Rhipicephalus, Dermacentor, Haemaphysalis,* and *Ornithodoros.* | Human, Domestic and Wild Hosts | Italy | PCR | 99% | (Chisu, Loi et al. 2020) |  |
|  | *Amblyomma nuttalli* | White  rhinoceros | Kenya | Genome analysis |  | (Nardi, Olivieri et al. 2021) |  |
|  | *A. americanum* | Laboratory-reared and field-collected | USA | qRT-PCR |  | (Kumar, Sharma et al. 2021) |  |
|  | *A. americanum* | Field-collected | USA | Quantitative PCR assays  Species-specific TaqMan assays |  | (Hensley, Zambrano et al. 2021) |  |
|  | *Amblyomma, Argas, Haemaphysalis, Hyalomma, Ornithodoros,*and  *Rhipicephalus* | Animals and vegetation | Zambia | PCR, Sanger sequencing | 45.7% | (Kobayashi, Chatanga et al. 2021) |  |
|  | *Amblyomma, Haemaphysalis* | Vegetation | Thailand | Real-time PCR assays | 65.8% | (Trinachartvanit, Maneewong et al. 2018) |  |
|  | *Ixodes persulcatus* | Cattle | China | PCR, Sanger sequencing | 27.80% | (Chao, Castillo et al. 2021) |  |
| ***Francisella*- Like Endosymbiont (FLE)** | | | | | | |  |
|  | *D. andersoni* | Field-collected | USA | PCR |  | (Niebylski, Peacock et al. 1997) |  |
|  | *Dermacentor  hunteri*,  *D. nitens*, *Dermacentor  occidentalis*  *A. maculatum*, and  *Ornithodoros porcinus* | Vegetation | USA | PCR | 60-80% | (Scoles 2004) |  |
|  | *D. variabilis* | Vegetation | USA | Multitarget real-time PCRs |  | (Kugeler, Gurfield et al. 2005) |  |
|  | *A. dubitatum, D. nitens* and *Rh. microplus* | Field-collected | Brazil | PCR |  | (Machado-Ferreira, Piesman et al. 2009) |  |
|  | *Hy. marginatum, Hy. aegyptium,* and *Rh. sanguineus* | Field-collected | Bulgaria | PCR |  | (Ivanov, Mitkova et al. 2011) |  |
|  | *A. maculatum*  *D. andersoni*  *D. occidentalis*  *D. variabilis* | Laboratory-reared and field-collected | USA | Broad-Range PCR/ESI-MS | 100%  100%  99.3%  100% | (Rounds, Crowder et al. 2012) |  |
|  | *D. reticulatus* | Field-collected | Hungary | Conventional PCR |  | (Kreizinger, Hornok et al. 2013) |  |
|  | *A. maculatum* | Laboratory-reared | USA | Genome Sequencing/anlysis,  Illumina Hi-Seq 2500 |  | (Gerhart, Moses et al. 2016) |  |
|  | *Hyalomma, Amblyomma,* and *Dermacentor* | Laboratory-reared and field-collected |  | PCR, Sanger sequencing | 21% | (Bonnet, Binetruy et al. 2017, Duron, Binetruy et al. 2017) |  |
|  | *Hyalomma* sp.  *Hy. marginatum*  *Hy. excavatum*  *Hyalomma dromedarii*  *Hy.* *aegyptium*  *Hyalomma* *rufipes* | Camels, horses, tortoises, migratory birds | Israel | Standard PCR and real-time RT-PCR | 90.6% (overall)  84.6%  90.5%  89.8%  100%  90.4% | (Azagi, Klement et al. 2017) |  |
|  | Ornithodoros moubata | Laboratory reared | France | 16S rDNA amplicon sequencing analysis, Diagnostic PCR assays and Sanger sequencing, Real-time quantitative PCR | 45% | (Duron, Binetruy et al. 2017) | |
|  | *I. ricinus,* and  *D. reticulatus* | Field-collected | Slovakia | PCR-based methods | 47.9% | (Kazimírová, Hamšíková et al. 2018) |  |
|  | *A. maculatum* | Vegetation | Georgia | Ion Torrent PGM  qPCR assays |  | (Dasch, Ramaiah et al. 2019) |  |
|  | *D. variabilis* | Vegetation |  | 16S rRNA gene  PCR |  | (Travanty, Ponnusamy et al. 2019) |  |
|  | *Hyalomma dromedarii* | Camels | Saudi Arabia | PCR |  | (Elbir, Almathen et al. 2020) |  |
|  | *Hy. dromedarii* | Camels | UAE | PCR, Sanger sequencing | 5.8 % | (Perveen, Muzaffar et al. 2021) |  |
|  | *A. americanum* | Laboratory-reared and field-collected | USA | qRT-PCR |  | (Kumar, Sharma et al. 2021) |  |
|  | *A. maculatum* | Field-collected | USA | Quantitative PCR assays  Species-specific TaqMan assays |  | (Hensley, Zambrano et al. 2021) |  |
| ***Rickettsia*-Like Endosymbiont (RLE)** | | | | | | |  |
|  | *I. persulcatus* | Vegetation | Russia | PCR |  | (Eremeeva, Oliveira et al. 2006) |  |
|  | *Ixodes woodi* | Laboratory-reared | USA | PCR |  | (Kurtti, Palmer et al. 2002) |  |
|  | *Carios capensis* | Nests of brown pelicans | USA | PCR |  | (Mattila, Burkhardt et al. 2007) |  |
|  | *Ixodes pacificus*  *Ixodes scapularis* | Laboratory-reared and field-collected | USA | Broad-Range PCR/ESI-MS | 100%  100% (egg)  81% (larva)  90.5% (nymph)  100% (lab ticks)  77-98.9% (field ticks) | (Rounds, Crowder et al. 2012) |  |
|  | *D. silvarum* | Field-collected | China | Diagnostic PCR assays |  | (Liu, Li et al. 2013) |  |
|  | *D. silvarum* | Field-collected | China | Quantitative PCR |  | (Liu, Li et al. 2016) |  |
|  | *Argas, Ornithodoros, Amblyomma, Dermacentor, Haemaphysalis, Hyalomma, Ixodes,* and *Rhipicephalus* | Laboratory-reared and field-collected |  | PCR, Sanger sequencing | 55.6% | (Bonnet, Binetruy et al. 2017, Duron, Binetruy et al. 2017) |  |
|  | *Ha. tibetensis* | Vegetations | China | PCR, RT-qPCR | 100% | (Wang, Li et al. 2017) |  |
|  | O. moubata | Laboratory reared | France | 16S rDNA sequencing, Diagnostic PCR assays and Sanger sequencing, Real-time quantitative PCR |  | (Duron, Binetruy et al. 2017) | |
|  | *D. everestianus* | Sheep, yaks and horses. | China | PCR | 100% | (Li, Li et al. 2019) |  |
|  | *I. scapularis* | white-tailed deer | UK | PCR and qPCR |  | (Al-Khafaji, Armstrong et al. 2020) |  |
|  | I. ricinus | Field collected | Netherlands | 16S rRNA qPCR |  | (Krawczyk 2021) | |
| ***Wolbachia*- Like Endosymbiont (WLE)** | | | | | | |  |
|  | I. scapularis, Rh. sanguineus, Ha. longicornis, O. moubata | Laboratory reared |  | PCR |  | (Noda, Munderloh et al. 1997) |  |
|  | *I. ricinus* | Vegetation | Germany | PCR |  | (Hartelt, Oehme et al. 2004) | |
|  | *I. ricinus* | Sheep | Netherlands | PCR |  | (Tijsse-Klasen, Braks et al. 2011) |  |
|  | *A. americanum* | Vegetation | USA | PCR | 3.5% to 25% in females | (Zhang, Norris et al. 2011) |  |
|  | *I. ricinus* | Vegetation |  | PCR |  | (Plantard, Bouju-Albert et al. 2012) |  |
|  | *I. ricinus* | Vegetation | Slovakia | Real-time and single-step PCR | 14% | (Subramanian, Sekeyova et al. 2012) |  |
|  | *Rh. microplus* | Cattle | Côte d'Ivoire | Real-time PCR and standard PCR assays |  | (Ehounoud, Yao et al. 2016) |  |
|  | *Rh. microplus* | Elephant | Thailand | PCR |  | (Hirunkanokpun, Ahantarig et al. 2018) |  |
|  | *Hyalomma anatolicum a* and  *Rh. microplus* | Field-collected | Pakistan | PCR |  | (Bobo 2020) |  |
|  | *Hy. anatolicum* and  *Rh. microplus* | Livestock | Pakistan | 16S rRNA gene  PCR |  | (Adegoke, Kumar et al. 2020) |  |
|  | *I. scapularis* | Vegetation | Massachusetts | 16S rRNA gene  PCR |  | (Benson, Gawronski et al. 2004) |  |
|  | I. ricinus | Red squirrels | UK | PCR | 15.2% | (Luu, Palomar et al. 2021) | |
|  | I.ricinus | Field collected | Netherlands | 16S rRNA qPCR |  | (Krawczyk 2021) | |
|  | *Rh. sanguineus* | Dogs | Taiwan | PCR | 55.8 (nymph), 39.8 (female), and 44% (male) | (Chisu, Mura et al. 2021) |  |

**Reference**

Adegoke, A., D. Kumar, C. Bobo, M. I. Rashid, A. Z. Durrani, M. S. Sajid and S. Karim (2020). "Tick-borne pathogens shape the native microbiome within tick vectors." Microorganisms **8**(9): 1299.

Al-Deeb, M. A., D. Frangoulidis, M. C. Walter, D. Kömpf, S. F. Fischer, T. Petney and S. B. Muzaffar (2016). "Coxiella-like endosymbiont in argasid ticks (Ornithodoros muesebecki) from a Socotra Cormorant colony in Umm Al Quwain, United Arab Emirates." Ticks and tick-borne diseases **7**(1): 166-171.

Al-Khafaji, A. M., S. D. Armstrong, I. V. Boccazzi, S. Gaiarsa, A. Sinha, Z. Li, D. Sassera, C. K. Carlow, S. Epis and B. L. Makepeace (2020). "Rickettsia buchneri, symbiont of the deer tick Ixodes scapularis, can colonise the salivary glands of its host." Ticks and tick-borne diseases **11**(1): 101299.

Al-Khafaji, A. M., S. R. Clegg, A. C. Pinder, L. Luu, K. M. Hansford, F. Seelig, R. E. Dinnis, G. Margos, J. M. Medlock and E. J. Feil (2019). "Multi-locus sequence typing of Ixodes ricinus and its symbiont Candidatus Midichloria mitochondrii across Europe reveals evidence of local co-cladogenesis in Scotland." Ticks and tick-borne diseases **10**(1): 52-62.

Arthan, W., C. Sumrandee, S. Hirunkanokpun, S. Kitthawee, V. Baimai, W. Trinachartvanit and A. Ahantarig (2015). "Detection of Coxiella-like endosymbiont in Haemaphysalis tick in Thailand." Ticks and Tick-borne Diseases **6**(1): 63-68.

Azagi, T., E. Klement, G. Perlman, Y. Lustig, K. Y. Mumcuoglu, D. A. Apanaskevich and Y. Gottlieb (2017). "Francisella-like endosymbionts and Rickettsia species in local and imported Hyalomma ticks." Applied and environmental microbiology **83**(18): e01302-01317.

Ben-Yosef, M., A. Rot, M. Mahagna, E. Kapri, A. Behar and Y. Gottlieb (2020). "Coxiella-like endosymbiont of Rhipicephalus sanguineus is required for physiological processes during ontogeny." Frontiers in microbiology **11**: 493.

Beninati, T., M. Riegler, I.-M. E. Vilcins, L. Sacchi, R. McFadyen, M. Krockenberger, C. Bandi, S. L. O'Neill and N. Lo (2009). "Absence of the symbiont Candidatus Midichloria mitochondrii in the mitochondria of the tick Ixodes holocyclus." FEMS microbiology letters **299**(2): 241-247.

Benson, M. J., J. D. Gawronski, D. E. Eveleigh and D. R. Benson (2004). "Intracellular symbionts and other bacteria associated with deer ticks (Ixodes scapularis) from Nantucket and Wellfleet, Cape Cod, Massachusetts." Applied and environmental microbiology **70**(1): 616-620.

Bobo, C. G. (2020). "Molecular Characterization of Wolbachia and Its Impact on the Microbiome of Exotic and United States Ticks."

Bonnet, S. I., F. Binetruy, A. M. Hernández-Jarguín and O. Duron (2017). "The tick microbiome: why non-pathogenic microorganisms matter in tick biology and pathogen transmission." Frontiers in cellular and infection microbiology **7**: 236.

Budachetri, K., D. Kumar, G. Crispell, C. Beck, G. Dasch and S. Karim (2018). "The tick endosymbiont Candidatus Midichloria mitochondrii and selenoproteins are essential for the growth of Rickettsia parkeri in the Gulf Coast tick vector." Microbiome **6**(1): 1-15.

Buysse, M., O. Plantard, K. D. McCoy, O. Duron and C. Menard (2019). "Tissue localization of Coxiella-like endosymbionts in three European tick species through fluorescence in situ hybridization." Ticks and tick-borne diseases **10**(4): 798-804.

Chao, L.-L., C. T. Castillo and C.-M. Shih (2021). "Molecular detection and genetic identification of Wolbachia endosymbiont in Rhipicephalus sanguineus (Acari: Ixodidae) ticks of Taiwan." Experimental and Applied Acarology **83**(1): 115-130.

Chisu, V., F. Loi, C. Foxi, G. Chessa, G. Masu, S. Rolesu and G. Masala (2020). "Coexistence of tick-borne pathogens in ticks collected from their hosts in Sardinia: an update." Acta Parasitologica **65**(4): 999-1004.

Chisu, V., L. Mura, C. Foxi and G. Masala (2021). "Coxiellaceae in Ticks from Human, Domestic and Wild Hosts from Sardinia, Italy: High Diversity of Coxiella-like Endosymbionts." Acta Parasitologica **66**(2): 654-663.

Dasch, G. A., A. Ramaiah, Z. C. Holmes, M. L. Zambrano and T. B. Shirey (2019). Use of the Ion Torrent PGM for Determining the Genomic Sequences of Francisella and Coxiella-Like Endosymbionts and Rickettsia Directly from Hard Ticks. Contemporary Acarology, Springer**:** 1-35.

Dergousoff, S. J. and N. B. Chilton (2011). "Novel genotypes of Anaplasma bovis,“Candidatus Midichloria” sp. and Ignatzschineria sp. in the Rocky Mountain wood tick, Dermacentor andersoni." Veterinary Microbiology **150**(1-2): 100-106.

Duron, O. (2015). "The IS1111 insertion sequence used for detection of Coxiella burnetii is widespread in Coxiella-like endosymbionts of ticks." FEMS Microbiology Letters **362**(17).

Duron, O., F. Binetruy, V. Noël, J. Cremaschi, K. D. McCoy, C. Arnathau, O. Plantard, J. Goolsby, A. A. Pérez de León and D. J. Heylen (2017). "Evolutionary changes in symbiont community structure in ticks." Molecular ecology **26**(11): 2905-2921.

Ehounoud, C. B., K. P. Yao, M. Dahmani, Y. L. Achi, N. Amanzougaghene, A. Kacou N’Douba, J. D. N’Guessan, D. Raoult, F. Fenollar and O. Mediannikov (2016). "Multiple pathogens including potential new species in tick vectors in Côte d’Ivoire." PLoS neglected tropical diseases **10**(1): e0004367.

Elbir, H., F. Almathen and A. Elnahas (2020). "Low genetic diversity among Francisella-like endosymbionts within different genotypes of Hyalomma dromedarii ticks infesting camels in Saudi Arabia." Veterinary World **13**(7): 1462.

Epis, S., M. Mandrioli, M. Genchi, M. Montagna, L. Sacchi, D. Pistone and D. Sassera (2013). "Localization of the bacterial symbiont Candidatus Midichloria mitochondrii within the hard tick Ixodes ricinus by whole-mount FISH staining." Ticks and Tick-borne Diseases **4**(1-2): 39-45.

Epis, S., D. Sassera, T. Beninati, N. Lo, L. Beati, J. Piesman, L. Rinaldi, K. McCoy, A. Torina and L. Sacchi (2008). "Midichloria mitochondrii is widespread in hard ticks (Ixodidae) and resides in the mitochondria of phylogenetically diverse species." Parasitology **135**(4): 485-494.

Eremeeva, M. E., A. Oliveira, J. B. Robinson, N. Ribakova, N. K. Tokarevich and G. A. Dasch (2006). "Prevalence of bacterial agents in Ixodes persulcatus ticks from the Vologda Province of Russia." Annals of the New York Academy of Sciences **1078**(1): 291-298.

Gerhart, J. G., A. S. Moses and R. Raghavan (2016). "A Francisella-like endosymbiont in the Gulf Coast tick evolved from a mammalian pathogen." Scientific reports **6**(1): 1-6.

Gofton, A. W., C. L. Oskam, N. Lo, T. Beninati, H. Wei, V. McCarl, D. C. Murray, A. Paparini, T. L. Greay and A. J. Holmes (2015). "Inhibition of the endosymbiont “Candidatus Midichloria mitochondrii” during 16S rRNA gene profiling reveals potential pathogens in Ixodes ticks from Australia." Parasites & Vectors **8**(1): 1-11.

Gottlieb, Y., I. Lalzar and L. Klasson (2015). "Distinctive genome reduction rates revealed by genomic analyses of two Coxiella-like endosymbionts in ticks." Genome biology and evolution **7**(6): 1779-1796.

Guizzo, M. G., L. F. Parizi, R. D. Nunes, R. Schama, R. M. Albano, L. Tirloni, D. P. Oldiges, R. P. Vieira, W. H. C. Oliveira and M. d. S. Leite (2017). "A Coxiella mutualist symbiont is essential to the development of Rhipicephalus microplus." Scientific Reports **7**(1): 1-10.

Harrus, S., A. Perlman-Avrahami, K. Mumcuoglu, D. Morick, O. Eyal and G. Baneth (2011). "Molecular detection of Ehrlichia canis, Anaplasma bovis, Anaplasma platys, Candidatus Midichloria mitochondrii and Babesia canis vogeli in ticks from Israel." Clinical Microbiology and Infection **17**(3): 459-463.

Hartelt, K., R. Oehme, H. Frank, S. O. Brockmann, D. Hassler and P. Kimmig (2004). "Pathogens and symbionts in ticks: prevalence of Anaplasma phagocytophilum (Ehrlichia sp.), Wolbachia sp., Rickettsia sp., and Babesia sp. in Southern Germany." International Journal of Medical Microbiology Supplements **293**: 86-92.

Hensley, J. R., M. L. Zambrano, A. J. Williams-Newkirk and G. A. Dasch (2021). "Detection of Rickettsia Species, and Coxiella-Like and Francisella-Like Endosymbionts in Amblyomma americanum and Amblyomma maculatum from a Shared Field Site in Georgia, United States of America." Vector-Borne and Zoonotic Diseases.

Hirunkanokpun, S., A. Ahantarig, V. Baimai and W. Trinachartvanit (2018). "A new record of Wolbachia in the elephant ticks from Thailand." Science Asia **44**: 44-47.

Ivanov, I. N., N. Mitkova, A. L. Reye, J. M. Hübschen, R. S. Vatcheva-Dobrevska, E. G. Dobreva, T. V. Kantardjiev and C. P. Muller (2011). "Detection of new Francisella-like tick endosymbionts in Hyalomma spp. and Rhipicephalus spp.(Acari: Ixodidae) from Bulgaria." Applied and environmental microbiology **77**(15): 5562-5565.

Jourdain, E., O. Duron, S. Barry, D. González-Acuña and K. Sidi-Boumedine (2015). "Molecular methods routinely used to detect Coxiella burnetii in ticks cross-react with Coxiella-like bacteria." Infection Ecology & Epidemiology **5**(1): 29230.

Kazimírová, M., Z. Hamšíková, E. Špitalská, L. Minichová, L. Mahríková, R. Caban, H. Sprong, M. Fonville, L. Schnittger and E. Kocianová (2018). "Diverse tick-borne microorganisms identified in free-living ungulates in Slovakia." Parasites & vectors **11**(1): 1-18.

Khoo, J.-J., F.-S. Lim, F. Chen, W.-H. Phoon, C.-S. Khor, B. L. Pike, L.-Y. Chang and S. AbuBakar (2016). "Coxiella detection in ticks from wildlife and livestock in Malaysia." Vector-Borne and Zoonotic Diseases **16**(12): 744-751.

Kobayashi, T., E. Chatanga, Y. Qiu, M. Simuunza, M. Kajihara, B. M. Hang’ombe, Y. Eto, N. Saasa, A. Mori-Kajihara and E. Simulundu (2021). "Molecular Detection and Genotyping of Coxiella-Like Endosymbionts in Ticks Collected from Animals and Vegetation in Zambia." Pathogens **10**(6): 779.

Krawczyk, A. I. (2021). Questing microbioticks: Interactions of microbes, ticks, vertebrates, and the environment, Wageningen University.

Kreizinger, Z., S. Hornok, Á. Dán, S. Hresko, L. Makrai, T. Magyar, M. Bhide, K. Erdélyi, R. Hofmann-Lehmann and M. Gyuranecz (2013). "Prevalence of Francisella tularensis and Francisella-like endosymbionts in the tick population of Hungary and the genetic variability of Francisella-like agents." Vector-borne and Zoonotic Diseases **13**(3): 160-163.

Kugeler, K. J., N. Gurfield, J. G. Creek, K. S. Mahoney, J. L. Versage and J. M. Petersen (2005). "Discrimination between Francisella tularensis and Francisella-like endosymbionts when screening ticks by PCR." Applied and Environmental Microbiology **71**(11): 7594-7597.

Kumar, D., S. R. Sharma, A. Adegoke, A. Kennedy, H. C. Tuten, A. Y. Li and S. Karim (2021). "Recently evolved Francisella-like endosymbiont outcompetes an ancient and evolutionarily associated Coxiella-like endosymbiont in the lone star tick (Amblyomma americanum) linked to the Alpha-Gal Syndrome."

Kurtti, T. J., A. T. Palmer and J. H. Oliver (2002). "Rickettsiella-like bacteria in Ixodes woodi (Acari: Ixodidae)." Journal of medical entomology **39**(3): 534-540.

Lalzar, I., S. Harrus, K. Y. Mumcuoglu and Y. Gottlieb (2012). "Composition and seasonal variation of Rhipicephalus turanicus and Rhipicephalus sanguineus bacterial communities." Applied and environmental microbiology **78**(12): 4110-4116.

Li, N., S. Li, D. Wang, P. Yan, W. Wang, M. Li, Z. Yu and J. Liu (2019). "Characterization of the Rickettsia-like and Coxiella-like symbionts in the tick Dermacentor everestianus Hirst, 1926 (Acari: Ixodidae) from the Qinghai-Tibet Plateau." Systematic and Applied Acarology **24**(1): 106-117.

Liu, L., L. Li, J. Liu, Y. Hu, Z. Liu, L. Guo and J. Liu (2013). "Coinfection of Dermacentor silvarum Olenev (Acari: Ixodidae) by Coxiella-like, Arsenophonus-like, and Rickettsia-like symbionts." Applied and environmental microbiology **79**(7): 2450-2454.

Liu, L., L. Li, J. Liu, Z. Yu, X. Yang and J. Liu (2016). "Population dynamics of multiple symbionts in the hard tick, Dermacentor silvarum Olenev (Acari: Ixodidae)." Ticks and tick-borne diseases **7**(1): 188-192.

Luu, L., A. M. Palomar, G. Farrington, A.-K. Schilling, S. Premchand-Branker, J. McGarry, B. L. Makepeace, A. Meredith and L. Bell-Sakyi (2021). "Bacterial Pathogens and Symbionts Harboured by Ixodes ricinus Ticks Parasitising Red Squirrels in the United Kingdom." Pathogens **10**(4): 458.

Machado-Ferreira, E., J. Piesman, N. S. Zeidner and C. A. Soares (2009). "Francisella-like endosymbiont DNA and Francisella tularensis virulence-related genes in Brazilian ticks (Acari: Ixodidae)." Journal of medical entomology **46**(2): 369-374.

Machado-Ferreira, E., V. F. Vizzoni, E. Balsemão-Pires, L. Moerbeck, G. S. Gazeta, J. Piesman, C. M. Voloch and C. A. Soares (2016). "Coxiella symbionts are widespread into hard ticks." Parasitology Research **115**(12): 4691-4699.

Mariconti, M., S. Epis, L. Sacchi, M. Biggiogera, D. Sassera, M. Genchi, E. Alberti, M. Montagna, C. Bandi and C. Bazzocchi (2012). "A study on the presence of flagella in the order Rickettsiales: the case of ‘Candidatus Midichloria mitochondrii’." Microbiology **158**(7): 1677-1683.

Mattila, J. T., N. Y. Burkhardt, H. J. Hutcheson, U. G. Munderloh and T. J. Kurtti (2007). "Isolation of cell lines and a rickettsial endosymbiont from the soft tick Carios capensis (Acari: Argasidae: Ornithodorinae)." Journal of medical entomology **44**(6): 1091-1101.

Mediannikov, O., L. Ivanov, M. Nishikawa, R. Saito, Y. N. Sidelnikov, N. I. Zdanovskaya, I. V. Tarasevich and H. Suzuki (2003). "Molecular evidence of Coxiella‐like microorganism harbored by Haemaphysalis concinnae ticks in the Russian Far East." Annals of the New York Academy of Sciences **990**(1): 226-228.

Najm, N.-A., C. Silaghi, L. Bell-Sakyi, K. Pfister and L. M. F. Passos (2012). "Detection of bacteria related to Candidatus Midichloria mitochondrii in tick cell lines." Parasitology research **110**(1): 437-442.

Nardi, T., E. Olivieri, E. Kariuki, D. Sassera and M. Castelli (2021). "Sequence of a Coxiella endosymbiont of the tick Amblyomma nuttalli suggests a pattern of convergent genome reduction in the Coxiella genus." Genome biology and evolution **13**(1): evaa253.

Niebylski, M. L., M. G. Peacock, E. R. Fischer, S. F. Porcella and T. G. Schwan (1997). "Characterization of an endosymbiont infecting wood ticks, Dermacentor andersoni, as a member of the genus Francisella." Applied and Environmental Microbiology **63**(10): 3933-3940.

Noda, H., U. G. Munderloh and T. J. Kurtti (1997). "Endosymbionts of ticks and their relationship to Wolbachia spp. and tick-borne pathogens of humans and animals." Applied and environmental microbiology **63**(10): 3926-3932.

Olivieri, E., S. Epis, M. Castelli, I. V. Boccazzi, C. Romeo, A. Desirò, C. Bazzocchi, C. Bandi and D. Sassera (2019). "Tissue tropism and metabolic pathways of Midichloria mitochondrii suggest tissue-specific functions in the symbiosis with Ixodes ricinus." Ticks and tick-borne diseases **10**(5): 1070-1077.

Papa, A., K. Tsioka, A. Kontana, C. Papadopoulos and N. Giadinis (2017). "Bacterial pathogens and endosymbionts in ticks." Ticks and tick-borne diseases **8**(1): 31-35.

Perveen, N., S. B. Muzaffar and M. A. Al-Deeb (2021). "Four tick-borne microorganisms and their prevalence in Hyalomma ticks collected from livestock in United Arab Emirates." Pathogens **10**(8): 1005.

Plantard, O., A. Bouju-Albert, M.-A. Malard, A. Hermouet, G. Capron and H. Verheyden (2012). "Detection of Wolbachia in the tick Ixodes ricinus is due to the presence of the hymenoptera endoparasitoid Ixodiphagus hookeri." PLoS One **7**(1): e30692.

Raele, D. A., D. Galante, N. Pugliese, E. De Simone and M. A. Cafiero (2015). "Coxiella-like endosymbiont associated to the “Anatolian brown tick” Rhipicephalus bursa in Southern Italy." Microbes and infection **17**(11-12): 799-805.

Rahal, M., H. Medkour, A. Z. Diarra, I. Bitam, P. Parola and O. Mediannikov (2020). "Molecular identification and evaluation of Coxiella-like endosymbionts genetic diversity carried by cattle ticks in Algeria." Ticks and Tick-borne Diseases **11**(5): 101493.

Ramaiah, A. and G. A. Dasch (2018). "Genome sequence of Coxiella-like endosymbiont strain CLE-RmD, a bacterial agent in the cattle tick (Rhipicephalus microplus) Deutsch strain." Genome announcements **6**(13): e00003-00018.

Rounds, M. A., C. D. Crowder, H. E. Matthews, C. A. Philipson, G. A. Scoles, D. J. Ecker, S. E. Schutzer and M. W. Eshoo (2012). "Identification of endosymbionts in ticks by broad-range polymerase chain reaction and electrospray ionization mass spectrometry." Journal of medical entomology **49**(4): 843-850.

Sassera, D., T. Beninati, C. Bandi, E. A. Bouman, L. Sacchi, M. Fabbi and N. Lo (2006). "‘Candidatus Midichloria mitochondrii’, an endosymbiont of the tick Ixodes ricinus with a unique intramitochondrial lifestyle." International journal of systematic and evolutionary microbiology **56**(11): 2535-2540.

Sassera, D., N. Lo, E. A. Bouman, S. Epis, M. Mortarino and C. Bandi (2008). "“Candidatus Midichloria” endosymbionts bloom after the blood meal of the host, the hard tick Ixodes ricinus." Applied and environmental microbiology **74**(19): 6138-6140.

Scoles, G. A. (2004). "Phylogenetic analysis of the Francisella-like endosymbionts of Dermacentor ticks." Journal of medical entomology **41**(3): 277-286.

Seo, M.-G., S.-H. Lee, I.-O. Ouh, G. H. Lee, Y.-K. Goo, S. Kim, O.-D. Kwon and D. Kwak (2016). "Molecular detection and genotyping of Coxiella-like endosymbionts in ticks that infest horses in South Korea." PLoS One **11**(10): e0165784.

Smith, T. A., T. Driscoll, J. J. Gillespie and R. Raghavan (2015). "A Coxiella-like endosymbiont is a potential vitamin source for the Lone Star tick." Genome biology and evolution **7**(3): 831-838.

Špitalská, E., O. Sparagano, M. Stanko, K. Schwarzová, Z. Špitalský, Ľ. Škultéty and S. F. Havlíková (2018). "Diversity of Coxiella-like and Francisella-like endosymbionts, and Rickettsia spp., Coxiella burnetii as pathogens in the tick populations of Slovakia, Central Europe." Ticks and tick-borne diseases **9**(5): 1207-1211.

Subramanian, G., Z. Sekeyova, D. Raoult and O. Mediannikov (2012). "Multiple tick-associated bacteria in Ixodes ricinus from Slovakia." Ticks and tick-borne diseases **3**(5-6): 406-410.

Takhampunya, R., J. Sakolvaree, N. Chanarat, N. Youngdech, K. Phonjatturas, S. Promsathaporn, B. Tippayachai, W. Tachavarong, K. Srinoppawan and B. K. Poole-Smith (2021). "The Bacterial Community in Questing Ticks From Khao Yai National Park in Thailand." Frontiers in veterinary science **8**.

Tijsse-Klasen, E., M. Braks, E.-J. Scholte and H. Sprong (2011). "Parasites of vectors-Ixodiphagus hookeri and its Wolbachia symbionts in ticks in the Netherlands." Parasites & vectors **4**(1): 1-7.

Travanty, N. V., L. Ponnusamy, M. L. Kakumanu, W. L. Nicholson and C. S. Apperson (2019). "Diversity and structure of the bacterial microbiome of the American dog tick, Dermacentor variabilis, is dominated by the endosymbiont Francisella." Symbiosis **79**(3): 239-250.

Trinachartvanit, W., S. Maneewong, W. Kaenkan, P. Usananan, V. Baimai and A. Ahantarig (2018). "Coxiella-like bacteria in fowl ticks from Thailand." Parasites & vectors **11**(1): 1-6.

Trinachartvanit, W., P. Rakthong, V. Baimai and A. Ahantarig (2018). "Candidatus midichloria sp in a Rhipicephalus sanguineus sL nymphal tick collected from a cat in Thailand." Southeast Asian Journal of Tropical Medicine and Public Health **49**(2): 251-255.

Trinachartvanit, W., W. Wutha, W. Kaenkan, I.-a. Chelong, M. Bahakheeree, V. Baimai and A. Ahantarig (2019). "Co-infection with Coxiella-like bacteria and Babesia in goat ticks from southern Thailand." Southeast Asian Journal of Tropical Medicine and Public Health **50**(4): 643-650.

Wang, R., N. Li, J. Liu, T. Li, M. Liu, Z. Yu and J. Liu (2017). "Symbiont dynamics of the Tibetan tick Haemaphysalis tibetensis (Acari: Ixodidae)." Parasites & vectors **10**(1): 1-8.

Williams-Newkirk, A. J., L. A. Rowe, T. R. Mixson-Hayden and G. A. Dasch (2012). "Presence, genetic variability, and potential significance of “Candidatus Midichloria mitochondrii” in the lone star tick Amblyomma americanum." Experimental and applied acarology **58**(3): 291-300.

Zhang, X., D. E. Norris and J. L. Rasgon (2011). "Distribution and molecular characterization of Wolbachia endosymbionts and filarial nematodes in Maryland populations of the lone star tick (Amblyomma americanum)." FEMS microbiology ecology **77**(1): 50-56.
